# Automorphy as a self-organizing DPP-dependent process that translates patterns into mechanical programs during *Drosophila* embryogenesis

**DOI:** 10.1126/sciadv.adv0311

**Published:** 2025-06-27

**Authors:** Baptiste Tesson, Stéphane A. Vincent

**Affiliations:** ^1^ Polarity, Division and Morphogenesis, Institut Curie, PSL Research University, CNRS UMR 3215, INSERM U934, F-75248 Paris Cedex 05, France.; ^2^ Laboratoire de Biologie et Modélisation de la Cellule, Ecole Normale Supérieure de Lyon, CNRS, UMR 5239, Inserm, U1293, Université Claude Bernard Lyon 1, 46 allée d’Italie, F-69364 Lyon, France.

## Abstract

Morphogens provide developing tissues with positional information to ensure coherent morphogenesis. Bone morphogenetic proteins (BMPs) initially form a gradient to pattern the dorsal domains of the *Drosophila* embryo. Here, we show that the BMP homolog decapentaplegic (DPP) endows dorsal domains with specific mechanical programs to organize morphogenesis. These domains self-organize using high local DPP activities, a process we call automorphy. Automorphy is key to inducing specific morphological changes while being faithful to the initial positional information. The BMP morphogen therefore uses a series of automorphic events to translate each position into physical potentials that later produce a contractile amnioserosa and a dorsal epidermis displaying plasticity. Plasticity allows cell elongation in wild-type embryos, and perturbations of cellular patterns reveal its crucial role in adapting to mechanical constraints. We propose that gradient formation and automorphy constitute complementary processes that allow BMPs to act as a morphogen in the *Drosophila* embryo.

## Teaser

Automorphy gets the morph into the morphogen.

## INTRODUCTION

Morphogens are factors that control the spatial organization of tissues during development to generate specific shapes. The existence of these diffusible shape-generating factors was originally proposed by Turing in his 1952 paper (*1*) to explain how gradients of competing signals break the zygote symmetry to allow the diversification of cell types and the subsequent determination of anatomical structures. The key idea of this model is that the signaling activities decrease, as these signals travel through tissues, inducing different reactions from naïve cells so they adopt different mechanical characteristics. Wolpert (*2*) proposed an elegant version of the patterning of several fields of cells by a single morphogen in his famous French flag model where the signaling gradient induces naïve cells to differentiate into at least three cell types. The attractiveness of this model comes from its apparent simplicity, as it presents a two-dimensional (2D) solution at a unique time point. A number of studies have revealed that Sonic Hedgehog (SHH) can generate a French flag pattern during neural patterning of the neural tube and that it requires complex gene regulatory networks to be interpreted (*3*–*5*). So after 70 years, the morphogen model is still a central paradigm in biology regarding patterning, but the relationship between the activity of the morphogen and the mechanical regulation leading to morphogenesis remains to be characterized.

Bone morphogenetic proteins (BMPs) act as morphogens to pattern the dorsal part of both vertebrate and invertebrate embryos (*6*, *7*). Before the cellularization of the *Drosophila* embryo, the secreted molecule short gastrulation (Sog) is secreted ventrally and diffuses toward the dorsal part of the embryo (*8*). It traps and inhibits two BMP homologs—decapentaplegic (DPP) and screw (Scw) (*9*)—and displaces them dorsally where they are released by the metalloprotease tolloid (*10*, *11*). This mode of action fits Turing’s prediction, as several diffusible molecules compete to establish a positional information gradient. This action generates two BMP gradients: first, a shallow one that represses the ventral neurogenic ectoderm fate and, later on, a second gradient that is narrowly positioned on the dorsal midline. This second gradient induces the marker *Zerknüllt* (*Zen*) that labels the dorsal-most tissue—the amnioserosa (*12*). Intermediate regions between the neurogenic ectoderm and the amnioserosa later express the GATA factor *pannier (pnr)* and develop into the dorsal epidermis (*13*, *14*). During the elongation of the trunk, Pnr specifies the dorsal epidermis (*15*) where it induces DPP, which then feeds back positively on *pnr* (*16*). This interaction yields a high accumulation of phosphorylated Mad, the transcription factor of the DPP pathway (*17*). At this stage, Zen represses DPP so the ligand is no longer expressed in the amnioserosa (*18*). There is therefore a biphasic activation of DPP with one high early activity that specifies the amnioserosa, followed by a second high activity that allows the formation of the dorsal epidermis (*17*, *19*). In summary, the BMP homologs DPP and Scw act as morphogens to pattern the dorsal part of the embryo, and the biphasic action of DPP leads to the formation of two tissues—the amnioserosa and the dorsal epidermis. At later stages, DPP is also secreted by the dorsal-most cells of the epidermis, at the time when dorsal tissues undergo the morphogenetic process of dorsal closure (*16*).

Dorsal closure is a key morphogenetic event of the late *Drosophila melanogaster* embryogenesis that exemplifies the control of morphogenesis by DPP (*20*–*25*). Specifically, at mid-embryogenesis, the embryo is left with a dorsal opening that is covered by the amnioserosa (*26*). By a complex interplay between myosin-dependent contraction, programmed cell death, and volume loss (*27*–*30*), the amnioserosa generates the main force that drives dorsal closure by pulling on the epidermis. In return, the epidermis elongates until it fuses at the midline (*31*, *32*). The late DPP is induced by c-Jun N-terminal kinase (JNK) signaling at the leading edge of the dorsal epidermis (*22*–*25*), and impairment of either JNK or DPP signaling induces a marked developmental failure. For example, mutants for the DPP receptor *tkv* (*16*) and the transcription factor *Jun Related Antigen (JRA)* (*23*, *24*, *33*) both display a dorsal-open phenotype where organs get extruded (*16*, *34*, *35*). Many reports proposed that leading-edge–expressed DPP induces morphogenesis: it would induce amnioserosa constriction and dorsal epidermis elongation (*21*, *36*). This implies a symmetric diffusion of DPP from the leading-edge cells to both the amnioserosa and the dorsal epidermis where it would reach about 10-cell diameters. In addition, late DPP activity fosters robust adhesion between the amnioserosa and the dorsal epidermis both basolaterally and through adherent junctions (*37*–*39*). So, dorsal closure appears to be an attractive model to understand the integration between signaling and morphogenesis.

In summary, the model is that the first two phases of BMP activities define the pattern of the tissues by specifying both the amnioserosa at stage 5 and the dorsal epidermis at stage 9, while the late, third phase of DPP activity from stage 11 coordinates the dynamics of closure by simultaneously inducing amnioserosa cell contraction, epidermis elongation, and the adhesion between both. Thus, this last wave of DPP is considered one of the finest examples of signaling-controlled morphogenesis by a diffusible signal (*21*).

Here, we attempted to use a holistic approach by dissecting the specific implications of the different DPP activities on the dynamics of morphogenesis. Unexpectedly, we find that amnioserosa cell contraction and dorsal epidermis cell elongation are independent of the third phase of DPP expression that emanates from the leading edge and correlates with the timing of morphogenesis. The data indicate that this late DPP activity is mainly required for the maintenance and strengthening of the adhesion between the amnioserosa and the dorsal epidermis. In addition, the data indicate that early BMP signaling, at stage 5, not only patterns the amnioserosa but also instructs its cells to become contractile. Next, the second phase of DPP signaling, at stage 9, patterns the dorsal epidermis and endows its cells to adopt a plastic behavior. This plasticity allows the embryo to adapt and compensate for strong tension forces, even when we markedly affect cellular patterns. So, instead of witnessing distinct DPP activities that control either patterning or morphogenesis, we observe that each phase of DPP expression controls the mechanical properties of a specific tissue by inducing high levels of signaling. Furthermore, DPP appears to act locally in the epidermis, thus remaining faithful to the positional information given by the initial BMP gradient. We define this local, self-organizing high signaling activity that triggers a mechanical program as “automorphy.” We propose that automorphy is a crucial step to translate positional information provided by the BMP morphogen gradient into specific mechanical programs. We discuss how intrinsic limits of DPP signaling to induce gene expression at low levels led to the selection of temporally regulated automorphic programs.

## RESULTS

### *Tkv* and *JRA* mutants display distinct phenotypes

**Fig. 1.**
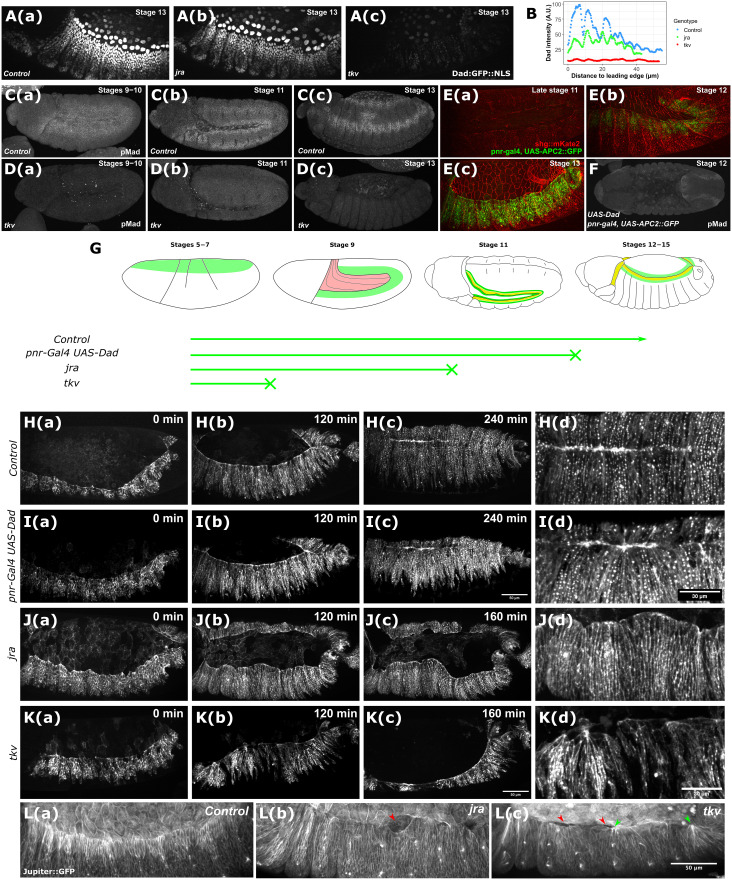
DPP signaling through development contributes later to dorsal closure. (**A**) Embryos expressing the Dad:GFP::NLS reporter in control (a), *jra* (b), or *tkv* (c) genetic background. (**B**) Dad:GFP::NLS reporter expression profile in the first abdominal segment of the embryos shown in (A) in arbitrary units (A.U.). (**C**) Phosphorylated Mad (pMad) stainings of stages 9 and 10 (a), stage 11 (b), or stage 13 (c) control embryos. (**D**) pMad stainings of stages 9 and 10 (a), stage 11 (b), or stage 13 (c) *tkv* mutant embryos. (**E**) Time-lapse imaging of shg::mKate2 embryos expressing the UAS_APC2::GFP reporter under the control of the pnr-Gal4 driver. (**F**) pMad staining of a stage 12 UAS-Dad pnr-Gal4 UAS-APC2::GFP embryo. (**G**) Schematics of the experimental procedure to selectively impair DPP signaling from different stages of development. Embryo schematics inspired from the Atlas of Drosophila Development (*67*). (**H** to **K**) Time-lapse imaging of embryos expressing the *UAS_APC2::GFP* reporter under the control of the *pnr-Gal4* driver in a control (H), *UAS-Dad* (I), *jra* (J), or *tkv* (K) background, from 0 to 240 min post–dorsal-closure onset for control and *UAS-Dad* embryos [(H), c and (I), c] or 160 min post–DC onset for *jra* and *tkv* embryos [(J), c and (K), c]. [(H) d, (I) d, (J) d, and (K) d] display close-ups from the end of closure (240 min) for control and Dad overexpression and midclosure (120 min) for *jra* and *tkv* mutants. Please note that by 120 min, the *tkv* embryo is already undergoing evisceration. (**L**) Close-up images of the leading edges of control (a), *jra* (b), and *tkv* (c) mutants in the *Jupiter::GFP* background. Red arrowheads indicate detachment between the amnioserosa and the dorsal epidermis, and green arrowheads indicate ipsilateral fusions of the dorsal epidermis.

To understand the action of the different phases of DPP on morphogenesis, we first characterized embryos of various genotypes to better define the action of DPP at specific stages of development. We first used *Dad-GFP* as a reporter of DPP activity to understand when *tkv* and *JRA* mutants affect the pathway (*40*, *41*). *Dad-GFP* is detected in the dorsal epidermis and the amnioserosa of stage 13 embryos, at the onset of dorsal closure [Fig. 1A (a)]. *Dad-GFP* is still detected in *JRA* null embryos in these tissues at this stage, indicating that JRA-induced DPP is not required for the expression of DPP targets at stage 13, either in the dorsal epidermis or in the amnioserosa [Fig. 1A (b)]. In contrast, both expressions are missing in *tkv* embryos of the same stage [Fig. 1, A (c) and B]. These data indicate that *JRA* and *tkv* do not affect DPP signaling at the same stage, which is intriguing as both mutants display a dorsal-open phenotype.

To verify that DPP signaling is affected earlier in *tkv* than in *JRA* embryos, we analyzed the phosphorylated state of Mad in controls and *tkv* embryos at various stages [Fig. 1, C (a) to D (c)]. The lack of phospho-mad detection in both the amnioserosa and the leading edge at stages 9 and 10 in *tkv* embryos confirms that DPP signaling is hampered way early in *tkv* mutants. On the other hand, JNK-dependent DPP induction at the leading edge is lacking in *JRA* mutants as shown by the absence of phospho-mad at stage 12 (fig. S1A). Therefore, phospho-mad detection confirms that DPP signaling is affected earlier in *tkv* than in *JRA* mutants. To obtain yet another tool to challenge DPP signaling, we overexpressed *Dad* in the pannier domain at stage 12 using *Adenomatous Polyposis Coli 2-GFP* as a marker (*Pnr-gal4 UAS-Dad, UAS-APC2-GFP*). The APC2–GFP (green fluorescent protein) fusion displays a robust fluorescence to accurately map the timing of overexpression. In addition, APC2-GFP overexpression was never found to be toxic (*42*). We observed that the *Pnr-Gal4* driver induces target gene expression from stage 12 onward [Fig. 1E, (a) to (c)], thus allowing us to express Dad after stage 11, when JNK induces DPP. We confirmed that Dad overexpression suppresses DPP activity at stage 12 (Fig. 1F). Figure 1G summarizes the duration of DPP activity during the development of the different genotypes. Together, these results indicate that DPP signaling stops earlier in *tkv* mutants than in *JRA* mutants.

We thus decided to monitor the development of *Pnr-Gal4 UAS-Dad* and *JRA* and *tkv* embryos using live imaging in a *Pnr-Gal4 UAS-APC2-GFP* background to visualize cell morphology in the dorsal epidermis [Fig. 1, H (a) to K (d), and movies S1 to S4]. Unexpectedly, Dad overexpression does not induce the dorsal-open phenotype but produces a scarring phenotype: the right and left leading edges do meet but the closure is not seamless, as cells from one side do not align with the cells from the other side [Fig. 1I, (a) to (d)]. This indicates that DPP is not required at the time of dorsal closure to prevent the dorsal-open phenotype. On the contrary, and as described in the literature, both *JRA* and *tkv* embryos end up dorsal open and eviscerated. The precise analysis of the onset of the evisceration indicates that a lack of adhesion between the epidermis and the amnioserosa is responsible for the dorsal-open phenotype. Live imaging reveals that in both *JRA* and *tkv* embryos, the epidermis progressively detaches from the amnioserosa [Fig. 1L, (a) and (c), and movies S5 to S7]. Still, differences are present, as *tkv* embryos display focal points where contiguous epidermal cells adhere in an ectopic manner [Fig. 1L (c)]. These ectopic fusions correlate with neighbor exchange at the leading edge, a feature that is common during wound healing across models (fig. S1B) (*43*–*46*). JNK signaling is high at the leading edge of *tkv* mutants (fig. S1C), and suppressing its activity with a dominant-negative form of JNK prevents the formation of the focal points, indicating that JNK activity is responsible for these ectopic adhesions [fig. S1, D to F, and movies S8 to S11]. Furthermore, the dorsoventral elongation in wild-type dorsal epidermal cells [Fig. 1H (d)] still occurs in *JRA* but appears limited in *tkv* embryos [Fig. 1, J (d) and K (d), and fig. S1, G and H]. Together, JRA and *tkv* zygotic mutants lose the capability to interpret DPP signaling at different stages and display distinct cellular phenotypes that still converge to produce the evisceration of the embryo.

### The amnioserosa still contracts in *tkv* embryos

**Fig. 2.**
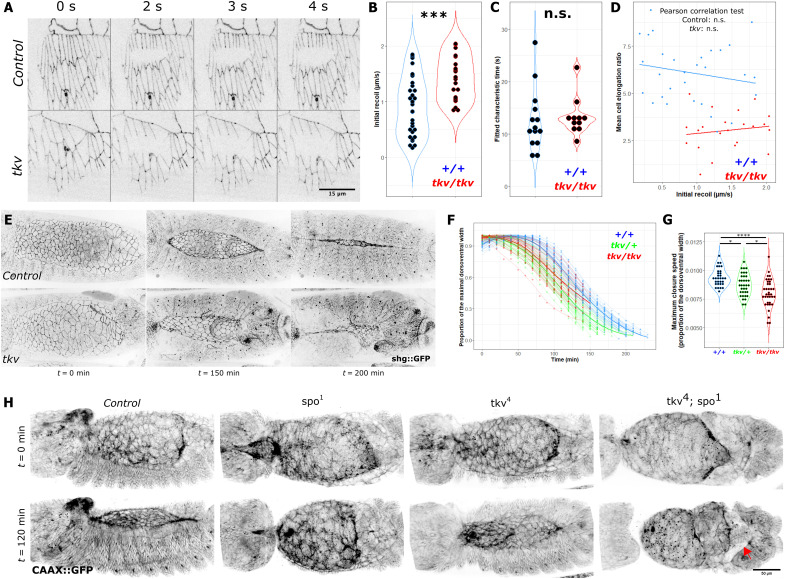
Amnioserosa contraction does not rely anymore on DPP signaling at the time of dorsal closure. (**A**) Maximum projection of time-lapse imaging of *shg::GFP* (H) and (H′) *tkv4/tkv4*; *shg::GFP* stage 14 embryos showing results of leading-edge laser ablation. (**B**) Comparison of the initial recoil velocity between *shg::GFP* (*n* = 30) and *tkv4/tkv4; shg::GFP* (*n* = 22) embryos among three technical replicates by Wilcoxon rank sum test. (**C**) Comparison of the characteristic time of retraction after ablation estimated by exponential fit between *shg::GFP* (*n* = 13) and *tkv4/tkv4; shg::GFP* (*n* = 11) embryos by Wilcoxon rank sum test. n.s., not significant. (**D**) Mean cell elongation of ablated cells as a function of initial recoil for *shg::GFP* (*n* = 30) and *tkv4/tkv4; shg::GFP* (*n* = 22). Linear regressions performed for each genotype are displayed as lines. (**E**) Time-lapse imaging of shg::GFP embryos in a control or *tkv4* genetic background. (**F**) Quantification of the amnioserosa short axis in function of time from the onset of DC for *shg::GFP* (*n* = 31), *tkv4/+; shg::GFP* (*n* = 35), and *tkv4/tkv4; shg::GFP* (*n* = 36) embryos among three technical replicates. LOESS (locally estimated scatterplot smoothing) regressions are performed for each embryo and displayed as lines. Mean LOESS regressions for each genotype are displayed as bold lines. Data are normalized by the maximum value reached by each embryo. (**G**) Comparison of the maximum short axis closure speed extracted from a five-parameter logistic regression fit for each amnioserosa closure of *shg::GFP* (*n* = 31), *tkv4/+; shg::GFP* (*n* = 35), and *tkv4/tkv4; shg::GFP* (*n* = 36) embryos. Comparison using analysis of variance (ANOVA) followed by Tukey post hoc tests. (**H**) Time-lapse imaging of CAAX::GFP embryos in a control, *tkv*, *spo*, or *tkv spo* background. Extruding hindgut in the *tkv spo* double mutant is indicated by a red arrow. *P < 0.05, ***P < 0.001, and ****P < 0.0001.

The deficiency in cellular elongation in *tkv* embryos could be due to a decrease in the pulling force generated by the amnioserosa. We thus used laser microsurgery in the dorsal epidermis to compare this tension between control and *tkv* embryos (Fig. 2A and movie S12). The cells of *tkv* embryos displayed a significantly higher initial recoil even if the values were strongly overlapping (Fig. 2B). However, because this discrepancy could be the consequence of a difference in stiffness or viscosity between the genotypes, we estimated the characteristic time of retraction in control and *tkv* embryos. This value is equal to the ratio between the stiffness and the viscosity in each genotype (Fig. 2C). Laser ablations revealed that the retraction time was similar between control and *tkv* embryos, indicating that the initial recoil is an accurate proxy for cell tension. Therefore, these results suggest that *tkv* embryos do not dissipate the energy from the pulling amnioserosa and controls. This is also supported by the observation that the expected total recoil after ablation estimated by exponential fit between control and *tkv* embryos is higher in *tkv* mutants (fig. S2A). Furthermore, while cells in *tkv* still elongate a bit, their elongation values overlap only with the lowest values measured in control embryos (Fig. 2D). Last, we did not notice any correlation between cell elongation and initial recoil (Fig. 2D). The absence of higher recoils in more elongated cells indicates that the gradual elongation of the dorsal epidermis does not result from an increase in pulling force over time generated by the amnioserosa, as suggested by earlier studies (*27*).

Next, we analyzed the dynamics of closure in terms of overall area (fig. S2B) and the distance separating the leading edge from the midline (Fig. 2E; see Materials and Methods and movie S13). While, on average, control embryos close slightly faster, the speed of the populations of embryos—wild-type, heterozygous, or homozygous—for *tkv* displays strong overlap, thus indicating that the dorsal-open phenotype does not result from a slower closure (Fig. 2, F and G, and fig. S2, B and C). These results indicate that the amnioserosa generates a pulling force and displaces the dorsal epidermis in *tkv* embryos. To further demonstrate the absence of the requirement of DPP signaling during amnioserosa contraction, we induced the DPP signaling antagonist Brk in the amnioserosa using *c381-Gal4* (fig. S2D and movie S14). While affecting zippering, this did not affect amnioserosa contraction, thus further confirming results from the Brunner Lab obtained by overexpressing Dad in the amnioserosa (*47*).

We next compared the *tkv* phenotype with the phenotype of a mutant where the amnioserosa does not contract: *spo* embryos are deficient in AS contraction due to a deficit in ecdysone signaling (*48*). At 120 min after the end of germ band elongation, we do not detect any contraction of the amnioserosa in *spo* mutants, while the contraction is visible in *tkv* embryos (Fig. 2H and movie S15). The double mutant recapitulates both the absence of contraction and the evisceration, indicating that the amnioserosa separates from the epidermis even in the absence of a strong pulling force (Fig. 2H). Together, these results confirm that the dorsal-open phenotype is not due to a lack of AS contraction but rather stems from the loss of adhesion between the amnioserosa and the dorsal epidermis [Fig. 1, (b) and (c)—see red arrowheads—and fig. S1D]. Furthermore, these data indicate that once it has been induced by the BMP morphogen as the most dorsal tissue, the amnioserosa does not need later DPP activity to exert the traction forces that shape morphogenesis. We then focused on the cell elongation defects present in the dorsal epidermis of *tkv* embryos.

### Early DPP is important for the elongation of dorsal epidermal cells during morphogenesis

**Fig. 3.**
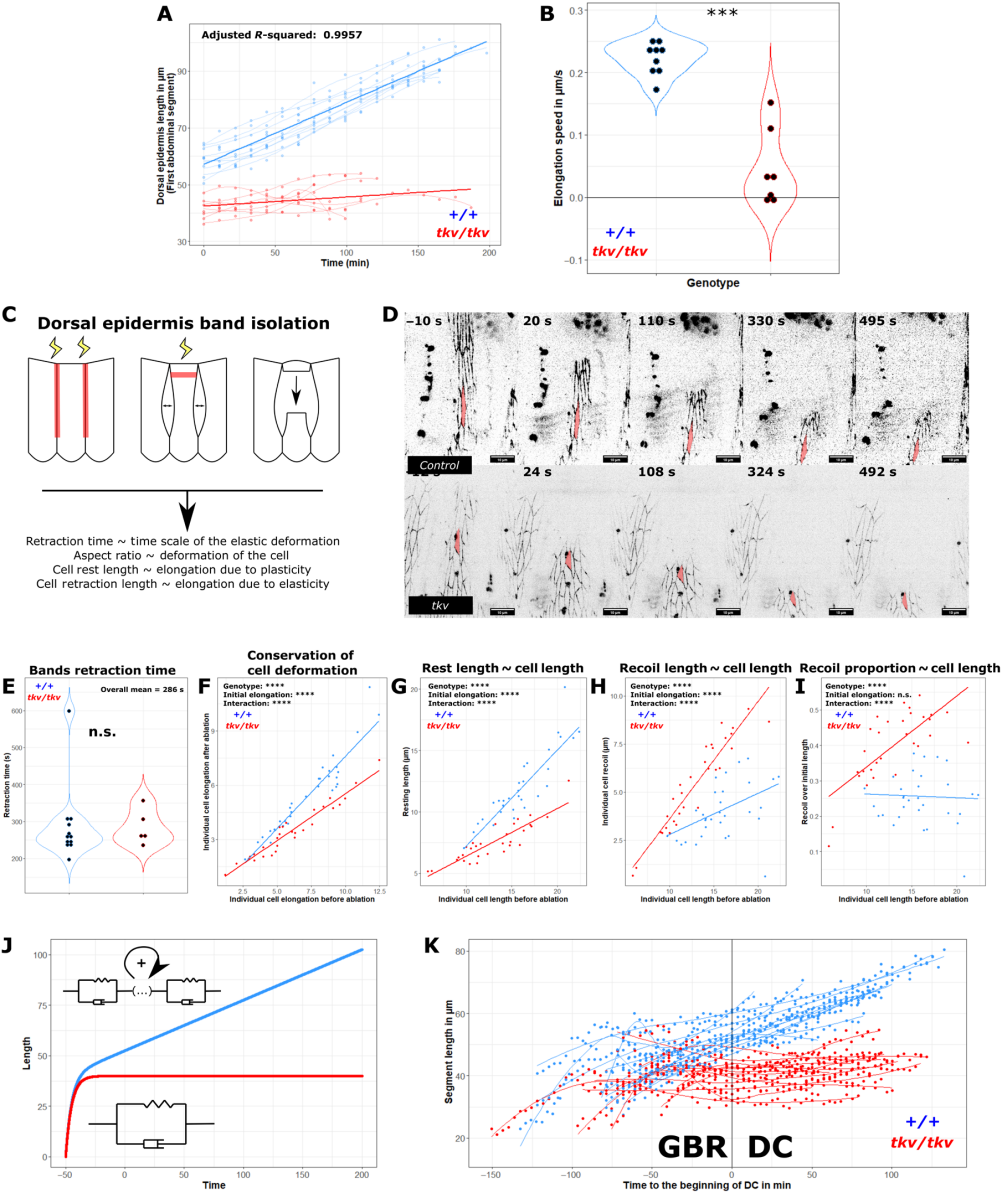
Early DPP controls dorsal epidermis elongation by inducing an elastic-to-plastic phase transition. (**A**) Elongation quantification in the A1 segment over time for *shg::GFP* (*n* = 9) and *tkv4 shg GFP* (*n* = 7). Simple lines: LOESS regression per embryo. Bold lines: Linear regression per genotype. (**B**) Elongation speed in *shg::GFP* (*n* = 9) and *tkv4 shg GFP* (*n* = 7) extracted by linear regression per embryo (adjusted *R*-squared: 0.9352). Comparison established with Wilcoxon test. (**C**) Experimental strategy for viscoelastic and viscoplastic property evaluation. Red lines indicate ablations. (**D**) Maximum projection of *shg::mKate2* and *tkv4 shg::mKate2* following (C). Red indicates segmented cells. (**E**) Retraction time of 12 stripes from *shg::mKate2* and 5 stripes from *tkv4 shg::mKate2*. Comparison established with Wilcoxon rank sum test. (**F** to **I**) Cell properties before and after retraction. A total of 33 cells from eight controls among two technical replicates and 30 tkv cells from seven stripes among three technical replicates. Comparison: Analysis of covariance (ANCOVA)’s *F* tests. Lines: Linear regression per genotype. (F) Cell elongation after retraction versus cell elongation before ablation. (G) Rest length of cells after retraction versus before ablation. (H) Retraction length of cells versus length before ablation. (I) Recoil over length before ablation as a function of length before ablation. (**J**) Simulation of elongation under constant force: Viscoelastic (red) and viscoplastic materials (blue). Viscoelastic characteristic time is estimated from (H) (5 min), maximal viscoelastic elongation from (J) (40 μm), and plastic elongation rate (speed at which new elastic units are added) from (B) (0.2 μm/min). (**K**) Elongation of abdominal segments of *shg::GFP; Jupiter::GFP* (*n* = 12 segments, six embryos) and *tkv4 shg::GFP; Jupiter::GFP* (*n* = 12 segments, four embryos) over time just after germ-band retraction (GBR). Lines: LOESS regression in each abdominal segment. ***P < 0.001 and ****P < 0.0001.

To characterize the elongation defects of *tkv* embryos, we first quantified the elongation of the epidermis in a dynamic manner using shg::GFP and focused on the first abdominal segment, as it is not compressed in *tkv* mutants. We took advantage of a specific dorsal bipolar neuron that develops at the ventral boundary of the *pnr* domain to measure the size of the dorsal epidermis (fig. S3). The control embryos display linear elongation during dorsal closure, from 60 to 90 μm (Fig. 3A). In comparison, the dorsal epidermis of *tkv* embryos is significantly less elongated from the onset of dorsal closure and displays little elongation until evisceration interrupts closure (Fig. 3A; evaluated from maximum segment length per embryo, Wilcoxon test). Speed estimation using individual linear regression for each elongation profile showed a significant decrease in *tkv* embryos (Fig. 3B): the mean elongation was reduced fivefold in *tkv* embryos, with five of seven *tkv* embryos barely showing any sign of elongation. Together, these results confirm that the elongation of the whole dorsal epidermis is markedly affected in *tkv* mutants, where DPP signaling is interrupted early in development. As elongation appears normal in *JRA* (Fig. 1J and fig. S1, G and H), where only late DPP signaling is affected, we can conclude that elongation is controlled by early DPP activity. As the second phase of DPP expression at stage 9 is active in the dorsal epidermis, we can conclude that this second phase of DPP specifies a mechanical program that underlies cell elongation during the later stages of morphogenesis.

### Early DPP endows cells with a plastic behavior

Next, we asked whether the elongation defects in *tkv* embryos stem from a nonspecific degeneration of the dorsal epidermis or whether they are due to the absence of a specific property that fails to be induced by DPP. We generated a 1D model to study the rheological properties of the dorsal epidermis; for details, see the “Rheological models of the dorsal epidermis” section in Materials and Methods. Linear elongation under constant traction could fit both a viscoelastic Kelvin-Voigt and a viscoplastic Maxwell-like model. In Kelvin-Voigt materials, the deformation dissipates upon stress release (see Eq. 3), whereas in Maxwell materials, deformation is partially conserved. To discriminate between these two models, we used laser ablation at the leading edge to release tension in a given segment after severing its segment boundaries to prevent lateral interference in both control and *tkv* embryos (Fig. 3, C and D, and movies S16 and S17). By 330 s, the recoil stops, and the elongation before ablation and after relaxation was measured for the cells that could be segmented (see the cell highlighted in red). A first indication came from the duration of the retraction, which happened within 6 min in either genotype. Under constant traction, Kelvin-Voigt materials elongate in a linear fashion only during a limited amount of time (for time, *t* ≪ τ, see Eq. 2), while in Maxwell materials, linear elongation is observed for higher time scales (see Eq. 5). Moreover, the characteristic viscoelastic elongation time is equal to its characteristic retraction time for both systems (see Eqs. 2 to 6). Quantification of the retraction time shows that the characteristic time of the viscoelastic deformation of the dorsal epidermis is inferior to 300 s for either genotype, about 40 times less than the duration of the process of dorsal closure itself in controls (Fig. 3E). Therefore, by reductio to absurdum, the dorsal epidermis does not behave as a purely viscoelastic Kelvin-Voigt material, as dorsal closure should last around 5 min under this assumption. To test the presence of a plastic behavior at the cellular level that would be lost in *tkv* mutants, we quantified the aspect ratio of cells within a band before and after release from the amnioserosa. While an important change in cell shape would advocate in favor of elasticity, shape conservation would argue for a plastic behavior. We removed from the analysis the cells exhibiting ectopic active constriction, a behavior reminiscent of a wound-healing response. We observed that initial elongation does contribute to cell elongation after relaxation in both genotypes; however, conservation of cell shape is greatly reduced in *tkv* compared to control embryos (Fig. 3F). Together, the results obtained both at the tissue and cellular level highlight the requirement of early DPP signaling for the plastic component that allows dorsal epidermis elongation during dorsal closure. To further characterize the properties of dorsal epidermal cells for both genotypes and highlight a DPP-dependent transition from Kelvin-Voigt to Maxwell material, we measured their length along the dorsoventral axis before and after relaxation (cells’ ellipsoid long axis after manual segmentation) and extracted the rest length and recoil length of each cell (Fig. 3, G and H). Quantification of rest length for both genotypes as a function of initial length confirmed the observation on cell shape conservation, as maintaining elongation implies higher rest length (Fig. 3G). However, an increase in cell length leads to higher recoil length for each genotype while being significantly higher in *tkv* mutants (Fig. 3H). These results confirmed the data from Fig. 2B suggesting that stress is stronger in *tkv* mutants compared to controls, thus leading to cell elongation in this case. However, this increase is not expected from a Maxwell material and suggests that both the increase in rest length and the stretching ability of the cells contribute to their elongation toward the midline in wild-type embryos. Papers describing cell plasticity show that it involves ratchet-like mechanisms at the molecular level and therefore the permanent addition or removal of cellular building units (*49*–*51*). To test whether this model would apply to our system, we measured the proportion of recoil as a function of initial cell length (Fig. 3I). In elastic Kelvin-Voigt–like material, the proportion would increase with cell length, while it would decrease in plastic Maxwell-like materials and stay constant in this intermediate model (see the “Rheological models of the dorsal epidermis” section in Materials and Methods). We observed that the proportion of recoil of control cells stays constant at around 25% in control embryos, which is compatible with the intermediate model. On the other hand, it increases from 25 to more than 50%, as cell length increases in *tkv* mutants, which is compatible with a Kelvin-Voigt material. Therefore, we built another plastic model, in which the dashpot contributing to plasticity adds new viscoelastic units in a ratchet-like manner (see Eq. 7). This stepwise addition could be either controlled by the amplitude of the stress, thus leading to a Maxwell-like elongation behavior, or, be constant, thus dissipating the traction at a constant rate *p* (see Eq. 8). We compared the dynamics of elongation under constant traction of this model to those in a Kelvin-Voigt model in Fig. 3J. We noticed that after a primary step of elastic behavior, the elastic model stops stretching to reach an asymptote, whereas the plastic model displays a linear increase in cell length over time. Both models share the primary step of pure elastic behavior. To check whether these elongation behaviors are recapitulated by live embryos, we monitored both control and *tkv* embryos from earlier stages (Fig. 3K). Notably, both genotypes display the initial elastic step during the stage of germ-band retraction that precedes dorsal closure. Still, only the cells from embryos with functional DPP signaling perform the linear, plastic elongation. This plastic behavior is absent in *tkv* embryos. We conclude that the second phase of DPP activity at stage 9 is instrumental in providing cells with the capability to adopt the plastic behavior that is critical during later morphogenesis.

### DPP-induced plasticity is key to morphogenesis

**Fig. 4.**
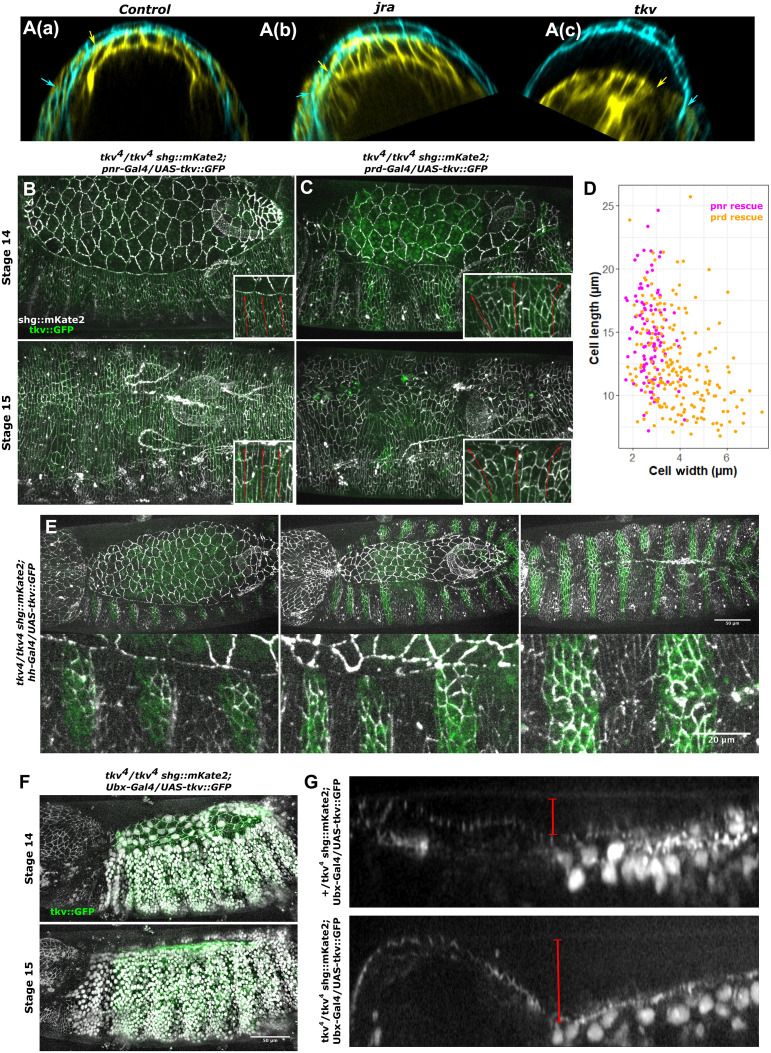
DPP-controlled cell plasticity allows adaptable and robust morphogenesis. (**A**) Transverse optical sections of time-lapse imaging of *CAAX::GFP* (a), *jra/jra; CAAX::GFP* (b), and *tkv4/tkv4; CAAX::GFP* (c) embryos. Cyan channels are stage 13 and yellow channels are stage 14 of the same embryo to appreciate changes in morphogenesis. Arrows of the corresponding color indicate the dorsal epidermis/amnioserosa junction. (**B**) Maximum of time-lapse imaging of *tkv4 shg::mKate2/tkv4; pnr-Gal4/UAS-tkv::GFP* from the onset of dorsal closure (stage 14) to its completion (stage 15) (*n* = 17 completion of closure, four technical replicates). Close-ups (bottom right) show the main orientation of cell elongation (red arrows). (**C**) Maximum projection of time-lapse imaging of *tkv4 shg::mKate2/tkv4; prd-Gal4/UAS-tkv::GFP* from the onset of closure (stage 14) to its completion (stage 15) (*n* = 7 completion of closure including 3 with anterior-open phenotype, two technical replicates). Close-ups (bottom right) show the main orientation of cell elongation (red arrows). (**D**) Quantification of cell long and short axes from ellipsoid fit after manual segmentation at closure completion in *shg::mKate2 tkv4/tkv4; pnr-Gal4/UAS-tkv::GFP* embryos (107 cells from five embryos) and *shg::mKate2 tkv4/tkv4; prd-Gal4/UAS-tkv::GFP* embryos (206 cells from four embryos). (**E**) Maximum projection of time-lapse imaging of *tkv4 shg::mKate2/tkv4; hh-Gal4/UAS-tkv::GFP* from the onset of closure to completion (*n* = 7 completion of DC and *n* = 10 dorsal-open phenotype, four technical replicates). (E) Associated close-ups on leading-edge cells. (**F**) Maximum of SD projection of time-lapse imaging of *tkv4 shg::mKate2/tkv4; Ubx-Gal4/UAS-tkv::GFP UAS-RFP::NLS* from the onset of closure (*n* = 18 completion of DC with anterior-open phenotype, four technical replicates). (**G**) Sagittal slices at closure completion associated with (F). Red bars indicate the distance between the epidermis at the boundary of the *Ubx* domain and the vitelline membrane seen by autofluorescence.

These results pose a conundrum: Why do we witness a displacement of the leading edge before evisceration in *tkv* embryos if their dorsal cells cannot stretch? To better understand this apparent contradiction, we analyzed transverse optical sections of embryos to visualize how their tissues behave tridimensionally during development (movie S18). In control and *JRA* embryos, where dorsal epidermal cells stretch, the tissues follow the curvature of the eggshell [Fig. 4A, (a) and (b)]. On the other hand, in *tkv* embryos, the dorsal epidermis, unable to undergo plastic deformation, dips into the embryo to accommodate the tension exerted by the amnioserosa [Fig. 4A (c)]. We conclude that the plastic elongation is critical for the epidermis to adapt to extrinsic tensions and to the tridimensionality of the embryo. Thus, the second phase of DPP at stage 9 induces a key morphogenetic program in the dorsal epidermis.

### Lack of DPP signaling in the mesoderm does not affect epidermal closure

The collapse of the dorsal epidermis in *tkv* embryos could be due to either the interplay of tensions or the modification of the structure or function of internal organs (*52*, *53*). To evaluate the impact of the absence of DPP signaling in internal organs on morphogenesis, we conducted a series of experiments. First, we analyzed the behavior of mesoderm-derived tissues in *tkv* embryos by expressing *UAS-APC2-GFP* under the control of *Twi-Gal4*. As previously published, the dorsal vessel does not form in embryos deficient in DPP signaling [(*52*); fig. S4A and movie S19]. Next, we rescued *tkv* function only in the mesoderm of *tkv* mutants to test whether restoring mesoderm dorsal specification would have an impact on the development of the ectoderm (fig. S4B and movie S20). In these embryos, evisceration was delayed compared to *tkv* mutants (fig. S4C). However, rescued embryos did not display any sign of elongation in the dorsal epidermis, indicating that the mesoderm phenotype of *tkv* embryos does not interfere with the morphogenesis of the dorsal epidermis (fig. S4D). Last, to observe specifically the effects of the absence of DPP signaling in the mesoderm while keeping the dorsal epidermis wild type, we overexpressed Brk in the mesoderm and monitored the behavior of the epidermis and amnioserosa using *shg-GFP* (fig. S4E and movie S21). We found that when only the mesoderm is unresponsive to DPP, the closure of the epidermis is complete and progresses with wild-type dynamics. Together, these data indicate that the lack of DPP signaling in the mesoderm does not interfere with the mechanics of epithelial closure. We can conclude that the cell elongation phenotype of *tkv* embryos is specific to the epidermis.

### Plasticity renders cells adaptable and allows robust morphogenesis

To explore the extent to which this mechanical program contributes to the adaptation of the dorsal epidermis to tension forces during morphogenesis, we modified the pattern and the number of cells that adopt a plastic behavior. We rescued *tkv* function in different subsets of cells in *tkv* mutant embryos (Fig. 4, B to F). As *pnr* expression is initiated in *tkv* embryos (*16*), we first tested the approach using the *pnr* driver that is specific to the dorsal epidermis and obtained a full rescue of dorsal closure (Fig. 4B and movie S22). To our surprise, the rescue driven in a *prd* pattern, which consists of only seven stripes along the embryo’s anteroposterior axis, also managed to save closure (Fig. 4C and movie S23). We next focused on the shape of the rescued cells in these two rescue experiments. First, we noticed that as in wild-type embryos, cells elongate along the dorsoventral axis when the full dorsal epidermis is rescued. On the other hand, cells stretch in multiple directions when only stripes of cells interpret DPP signaling (Fig. 4D). To further characterize whether the cells actively contribute to their deformation, we rescued *tkv* function in tighter bands, in the *hh* domain. Because the tissue does not perform neighbor exchange during dorsal closure, generating tighter bands does prevent rescued cells from elongating because their nonrescued neighbors lag behind (Fig. 4E and movie S24). These cells increase their cadherin levels at the apical junctions and expand their area and perimeter while markedly decreasing their aspect ratio compared to nonrescued *tkv* mutant cells. This suggests that DPP allows high tensions to trigger an increase in the apical surface of the cells, resulting in the adaptation of the tissue to the physical forces exerted by other tissues during morphogenesis. We conclude that the second phase of DPP signaling at stage 9 induces a mechanical program that controls cell plasticity to provide adaptability during morphogenesis.

### DPP defines a morphogenetic potential able to integrate multiple physical parameters

The cell-autonomous rescue of DPP signaling with *tkv* allows the comparison between adaptable and not adaptable regions at the anatomical level: We rescued *tkv* in the *Ubx* pattern so that only the abdominal segments adopt the plastic behavior (Fig. 4F and movie S25). We witnessed a rescue of dorsal closure and the failure of head involution, one of the final acts of embryonic morphogenesis. Head involution involves the cooperation between several organs that until then develop autonomously. The central nervous system moves inside the thoracic segments that themselves move anteriorly. When the head and the thorax are not rescued, the optic lobes stay outside of the embryo, and the first rescued segments elongate less toward the dorsal midline than in the control (Fig. 4G). This indicates that plasticity allows the dorsal epidermis to adapt to the volume of the internal organs of the embryo. Thus, DPP provides a morphological potential that is able to integrate the multiple parameters such as internal organ volume and embryonic surface, which ultimately define the final shape of the embryo.

Together, these data show that the second phase of DPP signaling instructs the dorsal epidermis to become an elongating tissue able to compensate for tension forces while robustly adapting to the volume of the visceral mass of the embryo. The action of the second phase of DPP at stage 9 mirrors the action of the first phase at stage 5 that specifies the amnioserosa as a tension generator. In addition, late DPP expression is dispensable for either elongation or contraction but maintains the adhesion between the amnioserosa and the dorsal epidermis. We conclude that the early BMP gradient instructs both positional information and the amnioserosa contraction program, while the second phase of DPP signaling instructs the plasticity program hours before morphogenesis. Both programs come into play later, at the time ecdysone induces morphogenesis.

## DISCUSSION

**Fig. 5.**
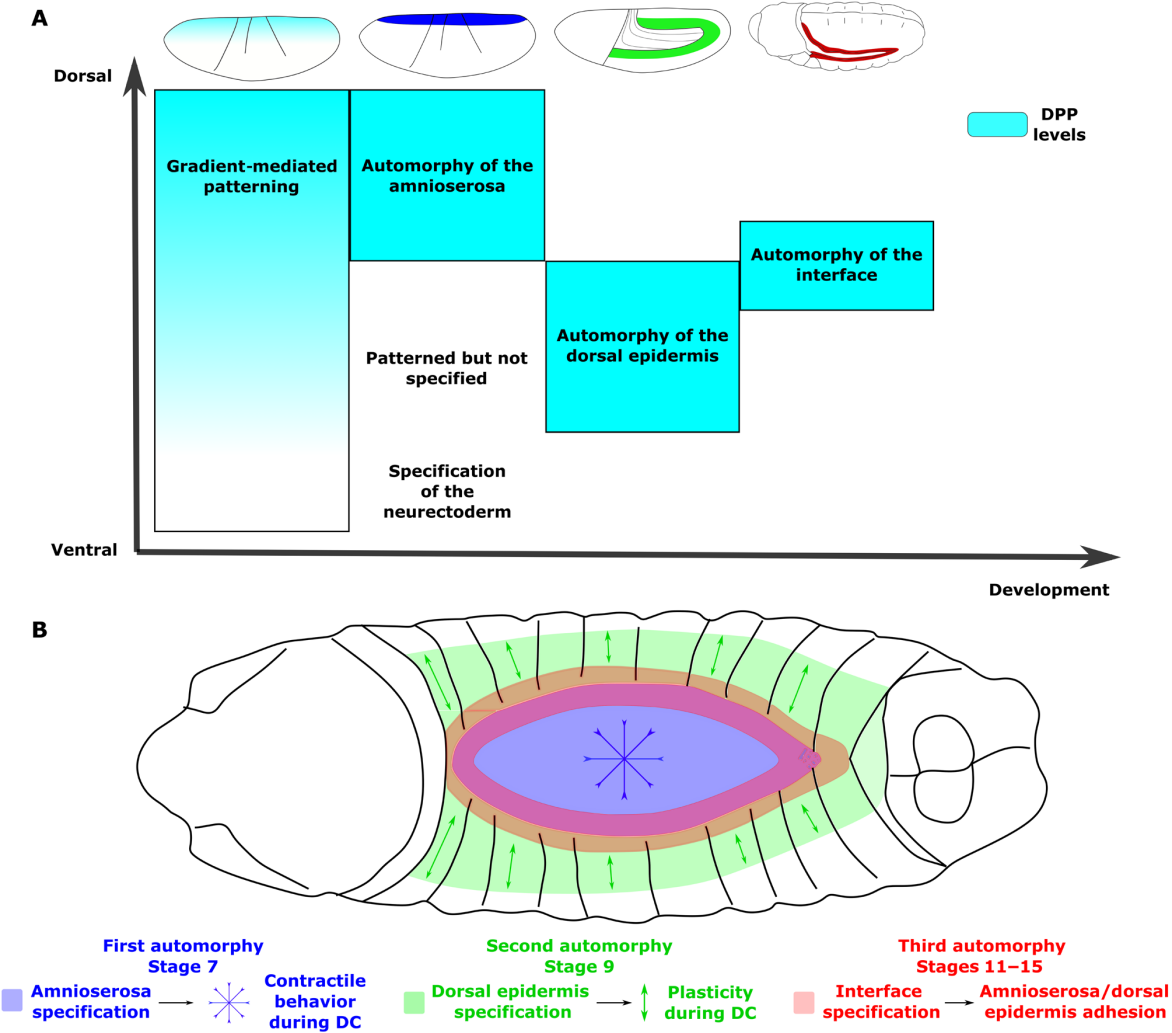
Automorphy translates DPP patterning into morphogenesis. (**A**) Timeline of DPP-mediated patterning and automorphic phases that leads to (**B**) a mechanical system that allows robust morphogenesis during dorsal closure. Cyan intensity indicates the strength of BMP/DPP signaling, and the other colors refer to the different automorphic phases (A) and the localization of the target tissues during morphogenesis (B).

The dynamic analysis of morphogenesis in embryos mutant for DPP or JNK pathways reveals that early phases of DPP signaling are sufficient for tissues to adopt specific mechanical properties later in development. These programs remain cryptic as long as the ecdysone hormone does not initiate morphogenesis [(*48*) and Fig. 2G]. These mechanical programs are controlled by high levels of DPP acting in a local manner and leading to their self-organization, a process we call automorphy, from ancient Greek αὐτο*-* (auto-, “self-”) and μορφή (morphḗ, “form”). Automorphy’s self-organization provides a simple strategy to remain faithful to the positional information set by the initial BMP morphogen and thus enables its gradient to be translated into complementary mechanical programs that provide coherence at the anatomical level (see Box 1). The control of dorsal morphogenesis by the BMP morphogen is schematized in Fig. 5 and can be summarized in the following way: First, the BMP gradient defines domains, with high signaling in the dorsal part of the gradient instructing cells to adopt later a contractile behavior. At later stages, the morphogen is reactivated in the original low signaling domain, but this time it triggers high signaling. The difference in outcome between the two domains stems from the change in cell competence, and this secondary phase of high signaling at stage 9 instructs cells to later adopt a plastic behavior. Automorphy is therefore the reactivation of morphogen signaling at high levels within a specific territory that leads to its self-organization and the instruction of specific mechanical properties (see Box 1). The sequential action of automorphic events therefore leads to the complementarity in mechanical properties with the contractile cells at the center of the initial gradient and the plastic cells directly surrounding them (Fig. 5B). Furthermore, the data indicate that the third phase of high DPP signaling, from stage 11, that acts during morphogenesis also uses automorphy to instruct cells to become adhesive so they can complete the morphogenetic process. Therefore, the morphogen organizes morphogenesis by acting sequentially: Initially, its gradient patterns territories, and next, during a succession of automorphic events, morphogenetic signaling resumes to instruct the cells within each territory so they adopt specific morphogenetic programs. The morphogen action is therefore not limited to gradient formation but involves intricate molecular mechanisms that span most of the development time. This double action, patterning then instruction, satisfies the paradigm proposed by Turing—that is, diffusible signals can both organize and control morphogenesis.

Box 1. **Presentation of automorphy.**

1) Automorphy defines a mechanical program that later translates into morphogenesis.

 2) Automorphy occurs in a specific region that has been defined at earlier developmental stages by a morphogen.

 3) Automorphy is initiated by a new phase of morphogen activity.

 4) This new phase of morphogen activity triggers high-level signaling, regardless of the strength of the original morphogen signal at that location.

 5) Gradient formation and automorphy are therefore the two processes that allow a morphogen to organize morphogenesis.

An unexpected outcome of this study is that during *Drosophila* embryogenesis, DPP-mediated automorphy selects the mechanical characteristics of the tissues but does not directly select the shape of the tissues during morphogenesis. The morphogen provides a morphogenetic potential by rendering cells adaptable. This adaptability is unexpectedly powerful as revealed by the experiments where we rescued *tkv* in subsets of cells. One important consequence of this plasticity is that the embryo is ready to put up with both tensions from the amnioserosa and the pressure from internal organs. It is therefore the integration of all these parameters that defines the ultimate shape of the embryo, indicating that automorphy provides a morphogenetic potential rather than a defined architecture.

The stage that best exemplifies the importance of automorphy is the acquisition of a plasticity program by the cells of the dorsal epidermis. The dorsal epidermis elongates in response to the contraction of the amnioserosa but little is known about the mechanism underlying this elongation, compared to the rich literature focusing on amnioserosa cell contraction (*27*–*30*). In this study, by probing tensions and tissue reactions with laser ablation, we show that DPP activity produces a phase transition selectively in the dorsal epidermis, thus turning the tissue from viscoelastic to viscoplastic. In addition, mathematical modeling predicts that *tkv* embryos should still display an elastic elongation at early stages, which was validated by studying the in vivo dynamics of these embryos at earlier stages during germ-band retraction. So, in *tkv* embryos, cells do not transition from a viscoelastic to viscoplastic behavior. Unexpectedly, this phase transition does not involve a jamming-to-unjamming transition as often seen in other models (*54*) but rather involves a transition at the cellular scale, a process that has been shown to rely on cadherin turnover (*49*–*51*). Our various rescue experiments, especially in the hh pattern, show that cadherin levels increase in rescued cells independently of cell deformation, thus suggesting that DPP increases cadherin levels at the junction to allow deformations. Thus, DPP signaling does not act by making the tissue flow through cell-neighboring exchanges but by getting the cells themselves to adopt a fluid behavior. This is exemplified by the expansion in cell apical surfaces in the direction of the prevailing tensions. We hypothesize that the absence of neighbor exchanges stems from the fact that the elongation of the dorsal epidermis during dorsal closure is transitory while unjamming results in the permanent deformation of tissues. Overall, this process is determinant for morphogenesis, as its absence leads to the collapse of the epidermis and its failure to accommodate the volume of the embryo. Elongation occurs in JNK embryos, indicating that the third phase of DPP, from stage 11 on, is dispensable for this transition. As the first phase at stage 5 is protected by the maternal effect, we can conclude that the second phase of DPP signaling at stage 9 triggers the plasticity program that is crucial for morphogenesis. Now that the dynamics of the process are better characterized, we can put these results in the perspective of the rich literature that describes the molecular interactions at the spatial level.

The second phase of DPP signaling at stage 9 occurs in the *pnr* domain, which corresponds to the positional information provided by the low BMP morphogen levels. *Pnr* transcription is not initiated by low DPP levels, as its enhancers do not display high-affinity Mad binding sites but are regulated by other factors, including the GAP genes (*55*). This explains why embryos lacking the Brk repressor and DPP and the mesoderm determinants *twi* and *snail* express *pnr* along the full dorsoventral axis in the middle of the anteroposterior axis (*56*). Thus, the Brk/DPP system is not required to induce *pnr* expression despite the fact that Brk prevents its expansion ventrally, setting the ventral limit of the *pnr* domain. These published results indicate that *pnr* is expressed by default in a domain that BMPs protect from the neurogenic ectoderm fate. This suggests that the weak BMP levels detected with phospho-Mad (*17*, *57*) do not need to induce transcription to define this domain and may not be able to induce a developmental program either. There is even a likely possibility that the dorsal epidermis is defined as the region where DPP and Brk perfectly cancel each other. This issue is resolved when *pnr* induces the second phase of DPP that is strong enough to initiate developmental programs. This robust expression is strengthened by the positive feedback from DPP on *pnr* (*16*). The induction of the mechanical program must respect the boundaries determined earlier by the BMP gradient, a crucial characteristic of automorphy. Automorphy therefore translates positional information into shape by the reiterative use of a higher activity of the same signal that still remains confined by the Brk antagonist that safeguards the ventral fate, keeping Brk as the guardian of positional information.

Another important information we obtained from the live analysis of *tkv* embryos is that canceling signaling after the initial BMP activity does not prevent amnioserosa contraction. Thus, the high BMP activity at cellularization not only defines the amnioserosa but is also sufficient for this tissue to contract. The expression pattern of *Zen*, the main amnioserosa marker, is highly dynamic: While its expression is first broad and independent of DPP, its maintenance and refinement in the dorsal domain during cellularization require DPP (*58*). During the first phase of refinement—that is, in the first half of cellularization—Brk must repress *Zen*, while this repression is no longer necessary later (*56*). This second, Brk-independent phase of refinement appears adequate for high DPP levels to induce the contraction program within the territory just defined by Brk. Thus, here also, automorphy is at work and uses a local, high level of DPP to self-organize the tissue and induce the contraction program of the amnioserosa.

Automorphy appears to also be involved in modifying the physical properties of the leading edge during the third phase of DPP signaling. JNK induces high levels of DPP, and both form a positive, “and”-type feed-forward motif. Thus, JNK induces DPP, and both signals together induce downstream targets (*20*). The target of autocrine DPP signaling, Zasp52, is important for the production of an actin cable that distributes tensions along the leading edge, thus increasing the robustness of the system (*59*). However, here, we demonstrate that in addition to the coherent feed-forward loop, DPP dampens JNK signaling and the JNK-induced wound-healing response to prevent ectopic fusions within a given leading edge. The territory corresponding to the leading edge is not encoded by a specific level of early BMP but is defined by the contact with the neighboring amnioserosa (*60*). Still, the same principle applies, and the program that defines the physical characteristics of these cells is also induced via an automorphic action of DPP. Thus, automorphy not only allows the enactment of morphogenesis in the territories patterned by the morphogen gradient but can also drive a physical behavior at the interface between two territories, which was not envisioned by the French flag model. Automorphy therefore provides a theoretical framework that better explains the observations made by researchers in the past few decades.

Together, we propose that automorphy is a self-organizing process mediated by a strong signal that defines a mechanical program. The reiterative use of the same signaling pathway allows automorphy to remain faithful to the boundaries set by the initial morphogen gradient. The activator-repressor system envisioned by Turing is therefore key to, first, establishing and, second, maintaining the spatial memory of the spatial information. The repressor is composed of two components—Sog that diffuses and shapes the BMP gradient early and the transcription repressor Brk that keeps mediating a negative feedback with DPP. The resulting positional information constrains automorphy so it generates mechanical diversity and ensures robust morphogenesis. A crucial aspect of the induction of the automorphic process is that the strong DPP signal does not directly rely on prior activation by the BMP pathway but recruits various regulatory components such as the GAP genes of the JNK pathway that are otherwise unrelated to the formation of the dorsoventral axis. This is explained by the fact that positional information can also be defined by an absence of signal and not only by inductions or repressions.

The morphogenetic potential that stems from the DPP induction of these mechanic programs may provide a key advantage for evolutionary innovation, as the adaptable tissue may welcome various modifications in shape during late embryogenesis. Accordingly, the gene regulatory network that enables the formation of the BMP morphogen gradient is conserved during evolution between invertebrates and vertebrates (*6*). It will be interesting to find out whether the BMP morphogen gradient confers adaptability in other species and whether it relies on automorphy to control the mechanics of development in vertebrates.

### Limitations of the study

While we show that DPP selects specific physical properties that the tissues display at the time of morphogenesis, we did not identify the downstream gene regulatory network that supports cytoskeletal modifications. While this does not affect our conclusions, it will be nice to understand the molecular basis of the viscoelastic to viscoplastic transition.

## MATERIALS AND METHODS

### Fly strains

Fly strains used were ${\mathrm{tkv}}^{4}$ (*61*), ${\mathrm{jra}}^{76-19}$ (BL #9880), *shg::GFP* (BL #60584), *shg::mKate2* (gift from Y. Bellaiche), *CAAX::GFP* (on II: Kyoto #109-824; on III: Kyoto #109-823), *Jupiter::GFP* (BL #6836), *TRE:GFP* (BL #59010), *Dad:GFP::NLS* (*62*), *UAS-APC2::GFP* (BL #8815), $\mathrm{UAS}-{\mathrm{Bsk}}^{\mathrm{DN}}$ (BL #6409), *UAS-tkv::GFP* (BL #51653), *Ubx-Gal4 UAS RFP::NLS* (gift from S. Merabet), *pnr-Gal4* (BL #3039), *prd-Gal4* (BL #1947), ${\mathrm{spo}}^{1}$ (BL #3276), and *UAS-brk* (gift from J. de Celis).

### Imaging

Crosses are kept at 25°C for at least 8 hours. Embryos are collected and dechorionated in 70% bleach, then washed, and aligned in Halocarbon oil 27 from Sigma-Aldrich.

### Laser ablation

Laser ablations are performed using a 355-nm pulsed laser on the spinning disk microscope. As the retraction of epithelia is known to follow the dynamics of Kelvin-Voigt materials, retraction profiles after ablation follow an exponential progression. Extracting multiple parameters from these profiles generates proxies for tissue properties: initial recoil for the ratio between tension and viscosity, characteristic time for the ratio between viscosity and stiffness, and, last, the total recoil for the ratio between tension and stiffness [as also shown in the “Rheological models of the dorsal epidermis” section in Materials and Methods and in (*63*)].

### Image analysis

Image analysis and measurements were performed using the Fiji software. Areas were measured in 2D from maximum projections, and the ellipsoid fit was performed in Fiji. Lengths were obtained from 3D measurements between neuron and leading edge and 3D positions in Fiji and calculated in R. Unless indicated otherwise, images displayed in the figures are maximum projections.

### Statistical analysis

All statistical tests were performed using R. Each test performed is indicated within the figure legends. Normality of the data and homoscedasticity were assessed using Kolmogorov-Smirnov and Fisher’s exact tests, respectively. Speeds were extracted from linear and logistic regressions; one regression is performed per embryo. Five-parameter logistic regression was performed using the *nplr* R package (*64*). Plots were generated using the *ggplot2* R package (*65*). Exponential fits were performed on Python using the SciPy library (*66*). ChatGPT was used occasionally to improve code efficiency and annotation.

### Rheological models of the dorsal epidermis

We considered three different models for the rheological response of the epidermis under constant traction σ.

We first consider the epidermis as a Kelvin-Voigt material, made of a spring (of Young modulus *k* and initial length $l_{0}$ ) and a dashpot (of viscosity $\eta_{\text{KV}}$ ) in parallel, therefore satisfying the equation

$$\sigma =k\left[l\left(t\right)-l_{0}\right]+\eta_{\text{KV}}\frac{\text{dl}\left(t\right)}{\text{dt}}$$ (1)

Therefore, under such condition, elongation under constant traction gives

$$l\left(t\right)=l_{0}+\frac{\sigma }{k}\left(1-e^{-\frac{t}{\tau }}\right)\text{with}\;\tau =\frac{\eta_{\text{KV}}}{k}$$ (2)

In this case, an increase of σ is required for l to increase once ≫τ . Moreover, we can also predict its behavior from the time traction is released

$$\begin{array}{c} l\left(t\right)=l_{0}+l_{\text{stretch}}e^{-\frac{t}{\tau }}\;\text{with}\;l_{\text{stretch}}=\frac{\sigma }{k}\left(1-e^{-\frac{t_{\text{release}}}{\tau }}\right) \end{array}$$ (3)

Therefore, for Kelvin-Voigt material, as considered for recoil experiment

$$\begin{array}{c} \text{Initial recoil}=l^{\prime }\left(0\right)=\frac{\sigma }{\eta_{\text{KV}}}\\ \text{Characteristic time}=\tau =\frac{\eta_{\text{KV}}}{k}\\ \text{Total recoil}=\underset{t\to \infty }{\text{lim}}l\left(t\right)=\frac{\sigma }{k}\;\text{for}\;t_{\text{release}}\gg \tau \end{array}$$

As our study of the elongation and release of the dorsal epidermis did not satisfy the properties of this model at the tissue level, we next updated it into a mixed Maxwell/Kelvin-Voigt model. We added a dashpot (of viscosity $\eta_{M}$ ) in series to the Kelvin-Voigt model previously described. Therefore in this model

$$\begin{array}{c} l\left(t\right)=l_{\text{dashpot}}\left(t\right)+l_{\text{Kelvin}-\text{Voigt}}\left(t\right)\\ \sigma =\sigma_{\text{dashpot}}=\sigma_{\text{Kelvin}-\text{Voigt}} \end{array}$$ (4)

Hence allowing to predict its elongation in response to constant traction as

$$l\left(t\right)=l_{0}+\frac{\sigma }{\eta_{M}}t+\frac{\sigma }{k}\left(1-e^{-\frac{t}{\tau }}\right)$$ (5)

and its behavior from the time traction is released as

$$\begin{array}{c} l\left(t\right)=l_{0}+l_{\text{deform}}+l_{\text{stretch}}e^{-\frac{t}{\tau }}\\ \text{with}l_{\text{deform}}=\frac{\sigma }{\eta_{M}}t_{\text{release}}\\ \text{and}l_{\text{stretch}}=\frac{\sigma }{k}\left(1-e^{-\frac{t_{\text{release}}}{\tau }}\right) \end{array}$$ (6)

As this second model did not satisfy the properties of elongation and release that we observed at the cellular level, we built this last model in which the epidermis is considered as a sum in a series of $n_{0}$ monomers behaving as Kelvin-Voigt materials. This suite of monomers grows in a ratchet-like manner, adding new monomers in response to constant traction σ linearly over time (similar to a dashpot of viscosity $\eta_{M}$ ). Therefore, under constant traction, this model satisfies

$$\left\{ \begin{array}{c} L\left(t\right)=\sum_{i=1}^{n_{\text{monomer}}}l_{{\text{monomer}}_{i}}\\ l_{{\text{monomer}}_{i}}=l_{0}+\frac{\sigma }{k}\left[1-e^{-\frac{\left(t-t_{i}\right)}{\tau }}\right]\\ n_{\text{monomer}}=\left\lfloor \frac{\sigma t}{l_{0}\eta_{M}}\right\rfloor \\ t_{i}=0\;\text{if}\;i<n_{0}\;\text{else}\;t_{i}=\left(i-n_{0}\right)\frac{l_{0}\eta_{M}}{\sigma } \end{array}\right.$$ (7)

which can be resolved as

$$\begin{array}{c} L\left(t\right)=n_{0}\left[l_{0}+\frac{\sigma }{k}\left(1-e^{-\frac{t}{\tau }}\right)\right]+\sum_{i=1}^{\left\lfloor \frac{t}{p}\right\rfloor }\left\{l_{0}+\frac{\sigma }{k}\left[1-e^{-\frac{\left(t-ip\right)}{\tau }}\right]\right\}\\ \text{with}\;p=\frac{l_{0}\eta_{M}}{\sigma } \end{array}$$ (8)

Thus introducing a polymerization period *p* to our model. Moreover, when the traction is released, the system satisfies

$$\begin{array}{c} L\left(t\right)=n_{0}l_{0}+L_{\text{deform}}+L_{\text{stretch}}e^{-\frac{t}{\tau }}\\ \text{with}\;L_{\text{deform}}=\left\lfloor \frac{t_{\text{release}}}{p}\right\rfloor l_{0}\\ \text{and}\;L_{\text{stretch}}=n_{0}\frac{\sigma }{k}\left(1-e^{-\frac{t_{\text{release}}}{\tau }}\right)+\sum_{i=1}^{\left\lfloor \frac{t_{\text{release}}}{p}\right\rfloor }\frac{\sigma }{k}\left[1-e^{-\frac{\left(t_{\text{release}}-ip\right)}{\tau }}\right] \end{array}$$

This model lastly satisfied all our observations, behaving as a Maxwell in series with a Kelvin-Voigt model during the elongation phase (as long as $n_{0}\gg \frac{\tau }{p}\gg 1$ ), while increasing both the amount of plastic and elastic deformation as the cells elongate.

To discriminate between the three models, we evaluated the proportion of cell recoil over total cell length as a function of cell length itself. For Kelvin-Voigt materials, cells increase their sizes, as $l_{\text{stretch}}$ increases, and $l_{0}$ is constant. Therefore, $\text{recoil ratio}=\frac{l_{\text{stretch}}}{l}=1-\frac{l_{0}}{l}$ is a function increasing with *l*.

For the mixed Kelvin-Voigt/Maxwell material, the traction σ is constant, therefore, $l_{\text{stretch}}$ is constant once t≫τ . Therefore, $\text{recoil ratio}=\frac{l_{\text{stretch}}}{l}$ is a function decreasing with l.

Last, in the polymerization model, $L_{\text{stretch}}\approx \frac{\sigma }{k}\left(n_{0}+\left\lfloor \frac{t_{\text{release}}}{p}\right\rfloor \right)$.

Therefore, $\begin{array}{c} \begin{array}{c} \text{recoil ratio}=\frac{L_{\text{stretch}}}{L}\approx \frac{\frac{\sigma }{k}\left(n_{0}+\left\lfloor \frac{t_{\text{release}}}{p}\right\rfloor \right)}{l_{0}\left(n_{0}+\left\lfloor \frac{t_{\text{release}}}{p}\right\rfloor \right)+\frac{\sigma }{k}\left(n_{0}+\left\lfloor \frac{t_{\text{release}}}{p}\right\rfloor \right)}=\frac{\frac{\sigma }{k}}{l_{0}+\frac{\sigma }{k}} \end{array} \end{array}$ , which is constant.

## Supplementary Materials

### The PDF file includes:

Figs. S1 to S4 Legends for movies S1 to S25 Legends for tables S1 to S14 Legends for codes S1 and S2

### Other Supplementary Material for this manuscript includes the following:

Tables S1 to S14

Codes S1 and S2
