# Supplementary material for: Automorphy as a self-organizing DPP-dependent process that translates patterns into mechanical programs during Drosophila embryogenesis
Source: Sci Adv. 2025 Jun 27;11(26):eadv0311. doi: 10.1126/sciadv.adv0311 (PMC12204166; doi:10.1126/sciadv.adv0311)
Supplement: Supplementary file 1 — Figs. S1 to S4 Legends for movies S1 to S25 Legends for tables S1 to S14 Legends for codes S1 and S2 [file sciadv.adv0311_sm.pdf]

Science Advances
AAAS

Supplementary Materials for

Automorphy as a self-organizing DPP-dependent process that translates patterns into mechanical programs during *Drosophila* embryogenesis

Baptiste Tesson and Stéphane A. Vincent

Corresponding author: Stéphane A. Vincent, stephane.vincent11@ens-lyon.fr

Sci. Adv. **11**, eadv0311 (2025)
DOI: 10.1126/sciadv.adv0311

The PDF file includes:

Figs. S1 to S4
Legends for movies S1 to S25
Legends for tables S1 to S14
Legends for codes S1 and S2

Other Supplementary Material for this manuscript includes the following:

Movies S1 to S25
Tables S1 to S14
Codes S1 and S2

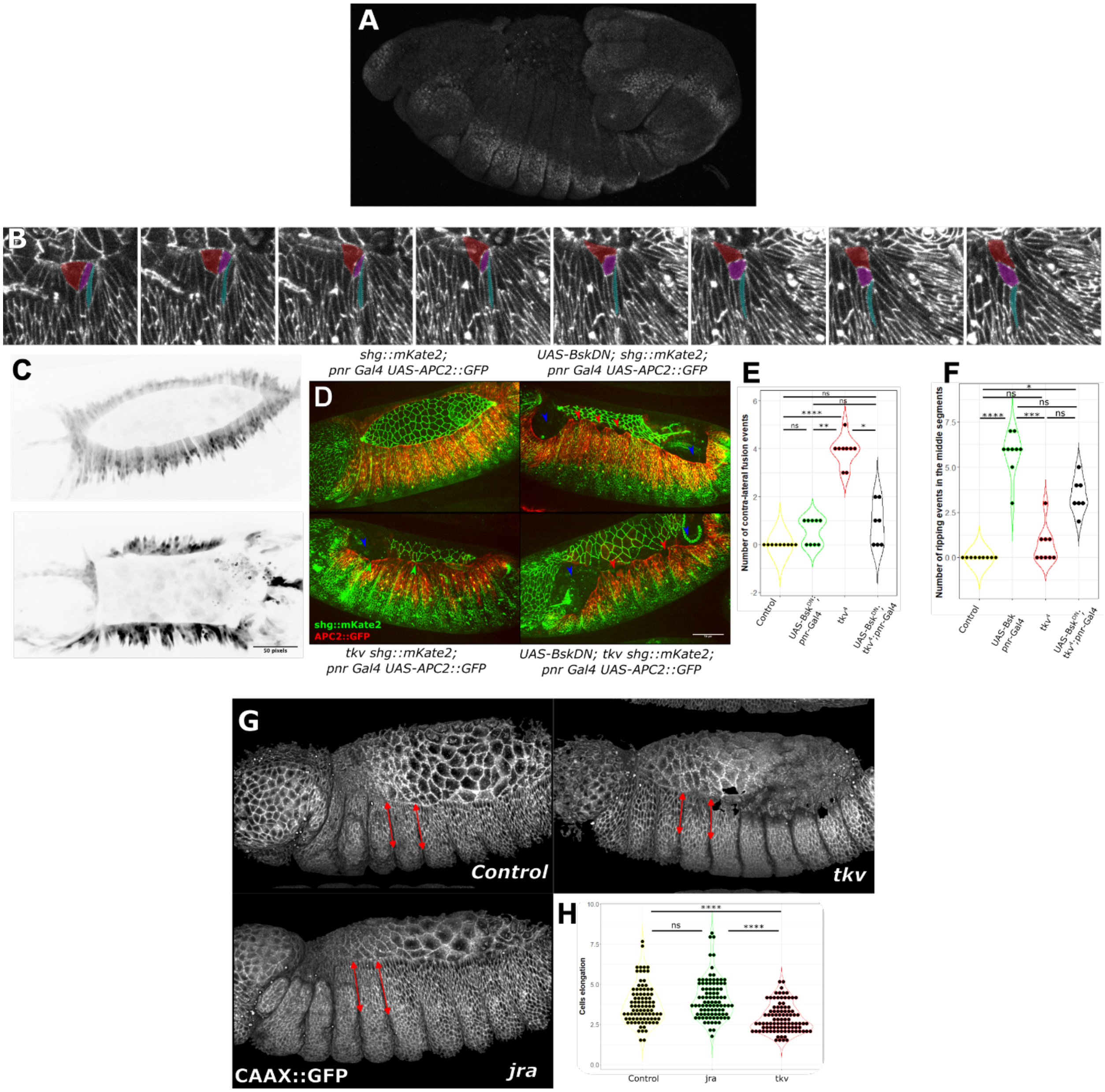

Fig. S1 : Defects in tkv and JRA mutants produce distinct phenotypes

A: pMad stainings of stage 12 *jra* mutant embryos related to Figure 1 C-D.

B: Time lapse imaging of the leading edge of an embryo in a *tkv shg::GFP* background. 3 cells are highlighted in order to show the intercalation at the leading edge, reminiscent of wound closure in the wild-type. Time step = 12 minutes.

C: Maximum projection of *TRE:GFP* (top), or tkv^8/tkv^8, *TRE:GFP* (bottom) at mid-dorsal closure

D: Maximum projection of *shg::mKate2; pnr Gal4 UAS-APC2::GFP, UAS-Bsk*^{DN}*; shg::mKate2; pnr Gal4 UAS-APC2::GFP , tkv*^{4}*/tkv*^{4} *shg::mKate2; pnr Gal4 UAS-APC2::GFP* and *UAS-Bsk*^{DN}*; tkv*^{4}*/tkv*^{4} *shg::mKate2; pnr Gal4 UAS-APC2::GFP* at mid-dorsal closure. Blue arrowheads indicate ripping of the epidermis from the anterior or posterior pole of the amnioserosa, red arrowheads indicate ripping of the epidermis from the amnioserosa from the middle segments, green arrowheads indicate ipsilateral leading edge fusion events.

E: Quantification of the number of ipsilateral fusion events observed in *pnr Gal4 UAS-APC2::GFP* (n=9), *UAS-Bsk*^{DN}*; shg::mKate2; pnr Gal4 UAS-APC2::*GFP (n=9), *tkv*^{4}*/tkv*^{4} *shg::mKate2; pnr Gal4 UAS-APC2::GFP* (n=9) and *UAS-Bsk*^{DN}*; tkv*^{4}*/tkv*^{4} *shg::mKate2; pnr Gal4 UAS-APC2::GFP* (n=7) among 5 technical replicates, compared by Kruskal-Wallis multiple comparison test, p-values adjusted with the Bonferroni method.

F: Quantification of the number of leading-edge detachment observed in *pnr Gal4 UAS-APC2::GFP* (n=9), *UAS-Bsk*^{DN}*; shg::mKate2; pnr Gal4 UAS-APC2::GFP* (n=9), *tkv*^{4}*/tkv*^{4} *shg::mKate2; pnr Gal4 UAS-APC2::GFP* (n=9) and *UAS-Bsk*^{DN}*; tkv*^{4}*/tkv*^{4} *shg::mKate2; pnr Gal4 UAS-APC2::GFP* (n=7) among 5 technical replicates, compared by Kruskal-Wallis multiple comparison test, p-values adjusted with the Bonferroni method.

G: Surface projections using the Surfcut2 Fiji plug-in of Control, *jra* and tkv embryos expressing the CAAX::GFP marker 60 minutes after the onset of dorsal closure. Dorsal epidermis domain of the first abdominal segment is indicated with red bars.

H: Quantification of cell elongation within the 4 first rows of cells of the first abdominal segment 60 minutes after the onset of dorsal closure of *Control CAAX::GFP* (89 cells among 4 embryos), *jra CAAX::GFP* (93 cells among 4 embryos) and *tkv* (95 cells among 4 embryos) **.** Comparison using ANOVA followed by Tukey HSD tests.

$*$: p−values < 0.05, $**$: p−values < 0.01, $***$: p−values < 0.001, $****$: p −values < 0.0001

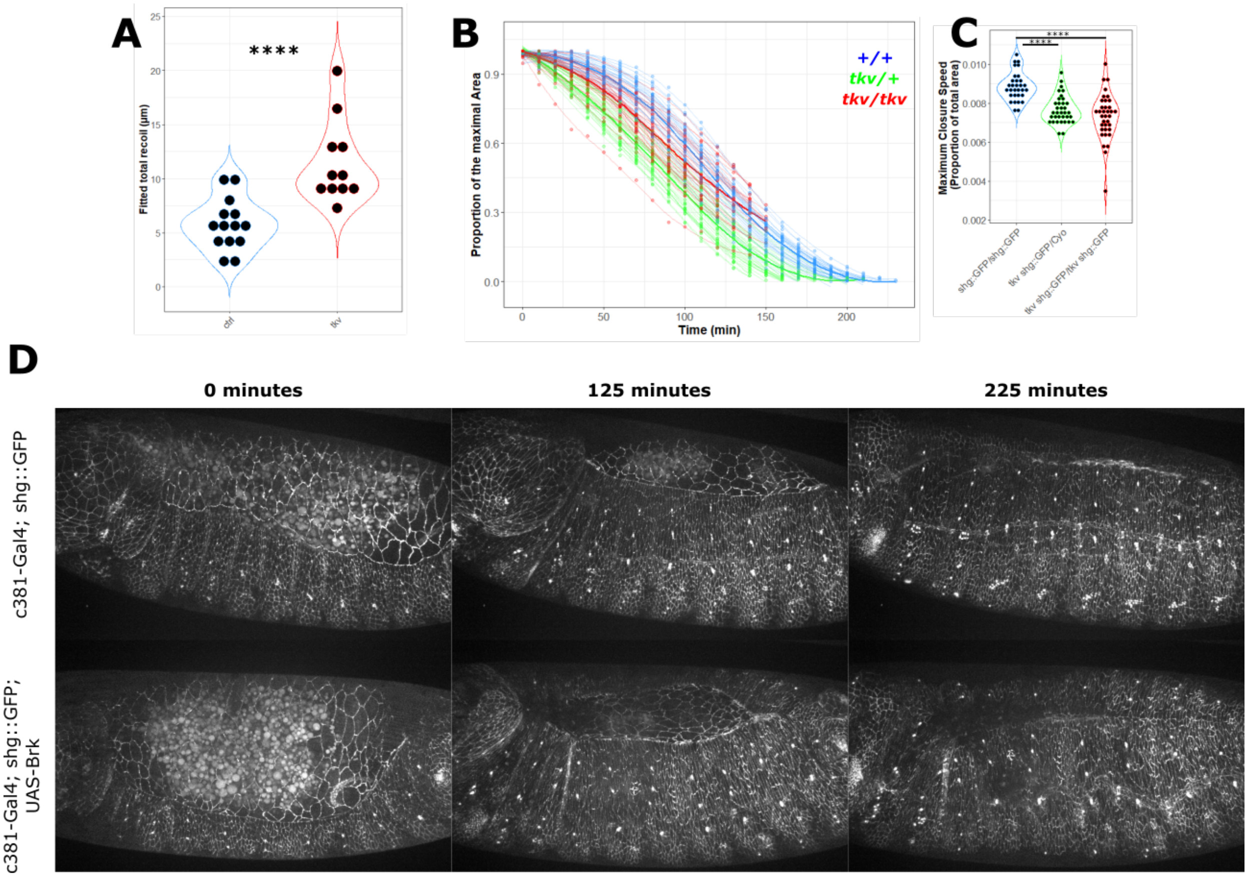

Fig. S2: Dynamic analysis of DPP function during dorsal closure

A: Comparison of the expected total recoil after ablation estimated by exponential fit between *shg::GFP* (n=13) and *tkv4/tkv4; shg::GFP* (n=11) embryos by Wilcoxon ranks-sum test.

B: Quantification of the amnioserosa area as a function of time from the onset of DC for *shg::GFP* (blue, n=31), $tkv^4/+$*; shg::GFP* (green, n=35) and tkv^4/tkv^4*; shg::GFP* (red, n=36) embryos among 3 technical replicates. Loess regressions are performed for each embryo and displayed as lines. Mean loess regressions for each genotype are displayed as bold lines

C: Comparison of the maximum area closure speed extracted from a 5-parameter logistic regression fit for each amnioserosa closure of *shg::GFP* (n=31), $tkv^4/+$*; shg::GFP* (n=35) and tkv^4/tkv^4*; shg::GFP* (n=36) embryos. Comparison using ANOVA followed by Tukey HSD tests.

D: Time-lapse imaging of *c381-Gal4; shg::GFP* and *c381-Gal4; shg::GFP ; UAS-Brk* embryos. Induction of Brk in the amnioserosa resulted in 10 scarred and one dorsal open embryos.
$*$: p−values < 0.05, $**$: p−values < 0.01, $***$: p−values < 0.001, $****$: p −values < 0.0001

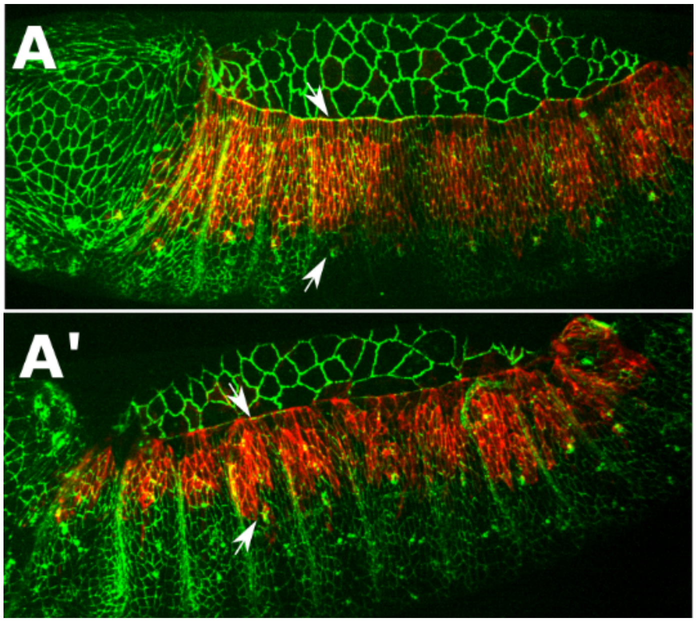

Fig. S3: Presentation of the genetic setup used to quantify the dorso-ventral dimension of the dorsal epidermis

Maximum projection of *shg::mKate2; pnr Gal4 UAS-APC2::GFP* (A) and *shg::mKate2 tkv4/tkv4; pnr Gal4 UAS-APC2::GFP* (A'), the third Bipolar neuron and leading edge used to measure the dorsal epidermis length are indicated by white arrows.

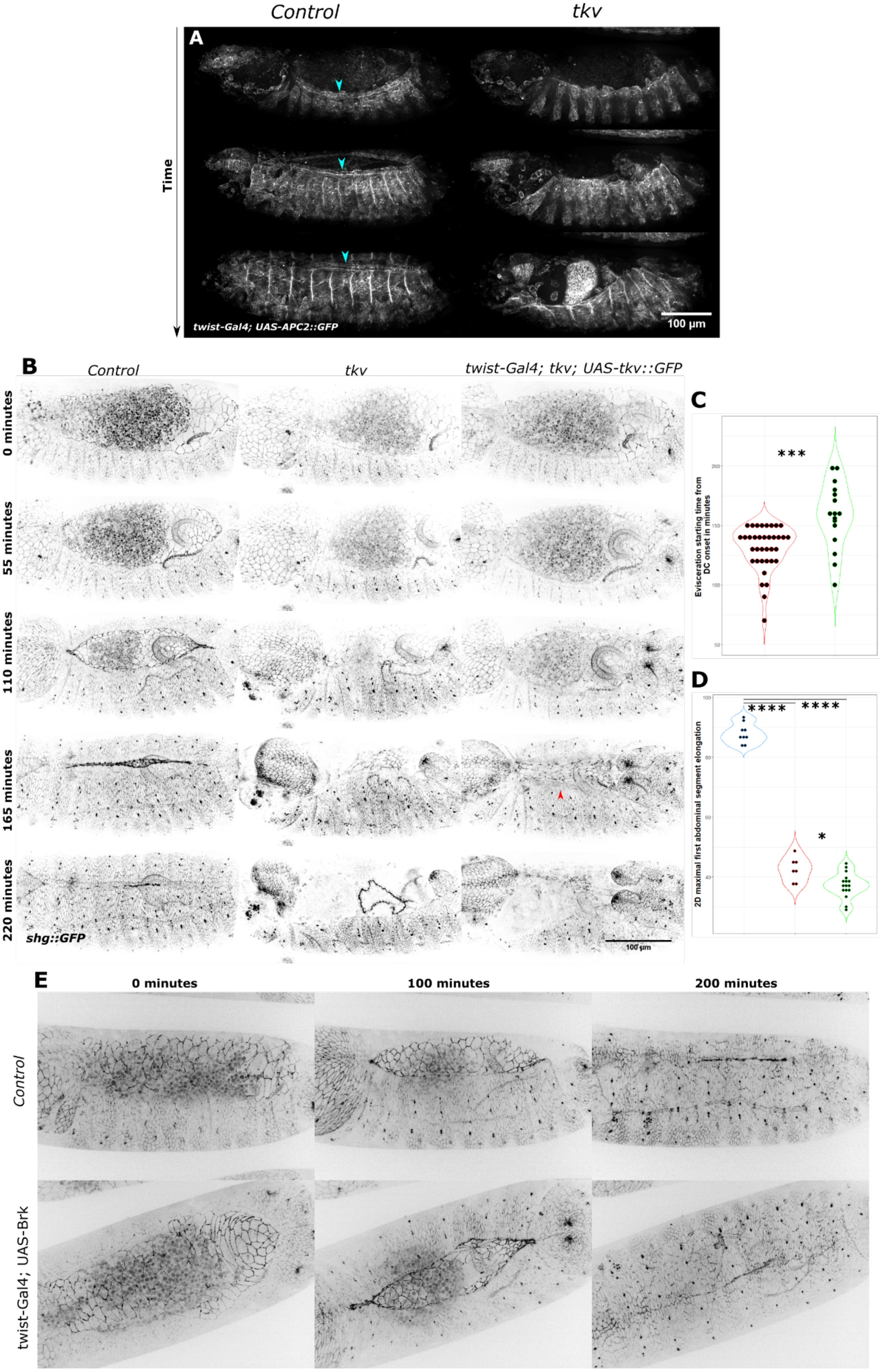

Control
tkv
A
Time
twist-Gal4; UAS-APC2::GFP
100 µm
B
Control
tkv
twist-Gal4; tkv; UAS-tkv::GFP
0 minutes
55 minutes
110 minutes
165 minutes
220 minutes
shg::GFP
100 µm
C
***
Evisceration starting time from DC onset in minutes
D
****
****
*
2D maximal first abdominal segment elongation
E
0 minutes
100 minutes
200 minutes
Control
twist-Gal4; UAS-Brk

Fig. S4: DPP action on the mesoderm does not impact the closure of the epidermis

A: Maximum projections of time-lapses from a *twist-Gal4UAS-APC2::GFP* and *twist-Gal4; tkv/tkv; UAS-APC2::GFP* embryos during dorsal closure. cyan arrowhead indicates the dorsal vessel in the control embryo.

B: Maximum projections of time-lapses from a *Control*, *tkv/tkv* and a *twist-Gal4; tkv/tkv; UAS-tkv::GFP* embryos during dorsal closure. Red arrowhead indicates the dorsal vessel in the rescued mutant.

C: Quantification of the evisceration starting time between *tkv* mutants and *tkv* mutants rescued in the twist domain (3 technical replicates). Evisceration times for *tkv* embryos were extracted from the same dataset as Fig 2E. Comparison carried using a Wilcoxon test.

D: Quantification of the projected distance as a proxy for total elongation of the first abdominal segment of *control, tkv* mutants and *tkv* mutants rescued in the twist domain (3 technical replicates). Elongation values for *control* and *tkv* were extracted from the same dataset as Fig 3A. Comparison carried by ANOVA followed by Tukey HSD test.

E: Maximum projections of time-lapses from a *Control*, *twist-Gal4; UAS-Brk* embryos during dorsal closure. All 3 *twist-Gal4; UAS-Brk* displayed no heart tube formation and no dorsal closure defects.
$*$: p−values < 0.05, $**$: p−values < 0.01, $***$: p−values < 0.001, $****$: p −values < 0.0001

Movie S1.
Time lapse imaging of an embryo expressing the *UAS-APC2::GFP* reporter under the control of the *pnr-Gal4* driver, t=0 indicates the onset of Dorsal Closure.

Movie S2.
Time lapse imaging of an embryo expressing the *UAS-APC2::GFP* reporter and *UAS-Dad* under the control of the *pnr-Gal4* driver, t=0 indicates the onset of Dorsal Closure.

Movie S3.
Time lapse imaging of an embryo expressing the *UAS-APC2::GFP* reporter under the control of the *pnr-Gal4* driver in *jra* background, t=0 indicates the onset of Dorsal Closure.

Movie S4.
Time lapse imaging of an embryo expressing the *UAS-APC2::GFP* reporter under the control of the *pnr-Gal4* driver in *tkv* background, t=0 indicates the onset of Dorsal Closure.

Movie S5.
Close up of time-lapse of the leading edges of a control embryo in the *Jupiter::GFP* background, , t=0 indicates the onset of Dorsal Closure.

Movie S6.
Close up of time-lapse of the leading edges of a *jra* embryo in the *Jupiter::GFP* background, , t=0 indicates the onset of Dorsal Closure.

Movie S7.
Close up of time-lapse of the leading edges of a *tkv* embryo in the *Jupiter::GFP* background, , t=0 indicates the onset of Dorsal Closure.

Movie S8.
Time lapse imaging of an embryo expressing the *UAS-APC2::GFP* reporter under the control of the *pnr-Gal4* driver in a *shg::mKate2* background.

Movie S9.
Time lapse imaging of an embryo expressing the *UAS-APC2::GFP* reporter and *UAS-Bsk*^{DN} under the control of the *pnr-Gal4* driver in a *shg::mKate2* background.

Movie S10.
Time lapse imaging of an embryo expressing the *UAS-APC2::GFP* reporter under the control of the *pnr-Gal4* driver in a *tkv shg::mKate2* background.

Movie S11.

Time lapse imaging of an embryo expressing the *UAS-APC2::GFP* reporter and *UAS-Bsk*^{DN} under the control of the *pnr-Gal4* driver in a *tkv shg::mKate2* background.

Movie S12.

Maximum projection of time-lapse imaging of *shg::GFP* (left) and *tkv*^{4}*/tkv*^{4}; *shg::GFP* (right) embryos showing the result of leading-edge laser ablation.

Movie S13.

Time lapse imaging of shg::GFP embryos in a control (top) or *tkv* (bottom) genetic background, t=0 indicates the onset of Dorsal Closure.

Movie S14.

Time-lapse imaging of *c381-Gal4; shg::GFP* (top) and *c381-Gal4; shg::GFP ; UAS-Brk* (bottom) embryos.

Movie S15.

Time lapse imaging of *CAAX::GFP* embryos in a *control* (top-left), *tkv* (top-right), *spo* (bottom-left) or *tkv, spo* (bottom-right) background, t=0 indicates the onset of Dorsal Closure.

Movie S16.

Maximum projection of time-lapse imaging of a *shg::mKate2* embryo during the experimental procedure described in Figure 3C.

Movie S17.

Maximum projection of time-lapse imaging of a *tkv shg::mKate2* embryo during the experimental procedure described in Figure 3C.

Movie S18.

Transverse optical section of time-lapse imaging of *CAAX::GFP* embryos in a *control* (top), *jra* (middle) and *tkv* background, t=0 indicates the onset of Dorsal Closure.

Movie S19.

Maximum projections of time-lapses from a *twist-Gal4UAS-APC2::GFP* and *twist-Gal4; tkv/tkv; UAS-APC2::GFP* embryos during dorsal closure.

Movie S20.

Maximum projections of time-lapses from a *Control*, *tkv/tkv* and a *twist-Gal4; tkv/tkv; UAS-tkv::GFP* embryos during dorsal closure.

Movie S21.

Maximum projections of time-lapses from a *Control*, *twist-Gal4; UAS-Brk* embryos during dorsal closure.

Movie S22.

Time lapse imaging of a partially rescued embryo expressing the *UAS-tkv::GFP* reporter under the control of the *pnr-Gal4* driver in a *tkv shg::mKate2* background, t=0 indicates the onset of Dorsal Closure.

Movie S23.

Time lapse imaging of a partially rescued embryo expressing the *UAS-tkv::GFP* reporter under the control of the *prd-Gal4* driver in a *tkv shg::mKate2* background, t=0 indicates the onset of Dorsal Closure.

Movie S24.

Time lapse imaging of a partially rescued embryo expressing the *UAS-tkv::GFP* reporter under the control of the *hh-Gal4* driver in a *tkv shg::mKate2* background, t=0 indicates the onset of Dorsal Closure.

Movie S25.

Time lapse imaging of a partially rescued embryo expressing the *UAS-tkv::GFP* and UAS-RFP::NLS reporters under the control of the *Ubx-Gal4* driver in a *tkv shg::mKate2* background, t=0 indicates the onset of Dorsal Closure.

Table S1.

Data associated to Figure 1B.

Table S2.

Data associated to Figure 2B.

Table S3.

Raw data associated to Figure 2C.

Table S4.

Processed data associated to Figure 2C.

Table S5.

Data associated to Figure 2D.

Table S6.

Data associated to Figure 3A.

Table S7.

Data associated to Figure 3E.

Table S8.

Data associated to Figure 3F-I.

Table S9.

Data associated to Figure 3K.

Table S10.

Data from embryos rescued using *pnr-Gal4* associated to Figure 4D.

Table S11.

Data from embryos rescued using *prd-Gal4* associated to Figure 4D.

Table S12.

Data associated so Figure S1 E-F.

Table S13.

Data associated so Figure S1 H.

Table S14.

Data associated so Figure S4 C-D.

Code S1.

R code used to generate all the data analysis and plots for this study.

Code S2.

Python code used to generate the exponential fit used for Figure 2C and S2A.
